# Supplementary material for: Improving deep learning protein monomer and complex structure prediction using DeepMSA2 with huge metagenomics data
Source: Nat Methods. 2024 Jan 2;21(2):279–89. doi: 10.1038/s41592-023-02130-4 (PMC10864179; doi:10.1038/s41592-023-02130-4)
Supplement: Supplementary file 1 — Supplementary Figs. 1–13, Tables 1–13 and discussion text 1 and 2. [file 41592_2023_2130_MOESM1_ESM.pdf]

nature methods

Article

https://doi.org/10.1038/s41592-023-02130-4

Improving deep learning protein monomer and complex structure prediction using DeepMSA2 with huge metagenomics data

In the format provided by the authors and unedited

Table of Contents

Supplement Figures

Figure S1. Head-to-head comparisons of the top 5*L* long-range mean absolute distance errors (*MAE*) between DeepMSA2 and (A) BLAST, (B) PSIBLAST, (C) MMseqs2, (D) HHblits, and (E) HMMER on CASP13, CASP14, and CASP15 monomer protein domains.

Figure S2. Comparisons of MSAs generated by DeepMSA2 and five control methods on 293 CASP monomer protein domains.

Figure S3. An illustration of (A) evolutionary information and (B) co-evolutionary information contained in multiple sequence alignments.

Figure S4. Case studies on domains from CASP13, CASP14, and CASP15, where the TM-scores of DMFold models have improved by more than 0.3 over those obtained using AlphaFold2.

Figure S5. Structural comparisons between DMFold models and AlphaFold2 DB models on 1,934 human proteins for which the DMFold creates high-quality models with pLDDT ⩾0.7, while AlphaFold2 DB models have a confidence score of pLDDT <0.7.

Figure S6. A head-to-head comparison between TM-score and pLDDT for final models by DMFold on 48 human proteins that have recently solved experimental structures, testing the performance of pLDDT as a binary classifier for whether a model is correctly folded.

Figure S7. Illustrative examples of H1140, H1141, and H1144 in the CASP15 Multimeric Modeling Section, which are all nanobody-antigen complexes.

Figure S8. Case study of target H1144 from the CASP15 Multimeric Modeling Section, which is a nanobody-antigen complex.

Figure S9. Case study of target H1142 from the CASP15 Multimeric Modeling Section, which is a nanobody-antigen complex.

Figure S10. TM-score of DMFold models versus (A) the *Neff* of DeepMSA2 MSAs, and (B) the alignment coverage between the query and homologous sequences of the DeepMSA2 MSAs on 62 CASP13, CASP14, and CASP15 'FM' monomer protein domains.

Figure S11. An illustration of the process used to generate paired MSAs in DeepMSA2-Multimer for a homomeric A3 complex (i.e., a homotrimer).

Figure S12. An illustration of the process used to generate paired MSAs in DeepMSA2-Multimer for a heteromeric A2B2C1 complex.

Figure S13. Architecture of DeepPotential networks for protein contact and distance map prediction.

Supplement Tables

Table S1. Benchmark results for the first threading template of HHsearch based on DeepMSA2's MSAs or the indicated third-party methods' MSAs on CASP13, CASP14, and CASP15 monomer domains.

Table S2. Long-range contact prediction precision by DeepPotential using MSAs from DeepMSA2 and the five control methods on CASP13, CASP14, and CASP15 monomer protein domains.

Table S3. Top 5*L* long-range mean absolute distance error (MAE) on CASP13, CASP14, and CASP15 monomer protein domains.

Table S4. Summary of the MSAs produced by DeepMSA2, BLAST, PSIBLAST, MMseqs2, HHblits, and HMMER on 293 CASP13, CASP14, and CASP15 monomer proteins.

Table S5. The average values of the number of effective sequences (*Neff*), number of homologous sequences (*Nseq*), and TM-scores of final models by three different programs on 132 FM domains in CASP13, CASP14 and CASP15.

Table S6. The average TM-score of final models produced by DMFold and AlphaFold2 DB on 48 human proteome proteins that have low AlphaFold2 DB pLDDT scores and recently solved experimental structures.

Table S7. The set of protein structures comprising our protein complex dataset, including 14 heteromer complexes and 40 homomer complexes from CASP13 and CASP14.

Table S8. The structure prediction ability of DMFold-Multimer and AlphaFold2-Multimer on 14 heteromer and 40 homomer complex targets collected from CASP13 and CASP14.

Table S9. Summary of the protein complex modeling results for all 87 participant groups in the CASP15 experiment.

Table S10. Comparison of the TM-score, LDDT, Interface Contact Score (ICS), and Interface Patch Score (IPS) results between DMFold-Multimer and AlphaFold2-Multimer in the CASP15 experiment.

Table S11. The monomer protein dataset used in our benchmark tests, including 48 free modeling (FM) domains and 64 template-based modeling (TBM) domains from CASP13, 37 FM domains and 50 TBM domains from CASP14, as well as 47 FM domains and 47 TBM domains from CASP15.

Table S12. Summary of genomic and metagenomics databases used in DeepMSA2.

Table S13. Impacts of BLAST filtering on the MSA generation step of the dMSA pipeline.

Supplement Texts

Text S1. DeepMSA2 provides balanced MSAs for monomer fold-recognition and spatial restraint prediction.

Text S2. Cases analyses of the CASP15 nanobody-antigen complexes.

References

Supplementary Figures

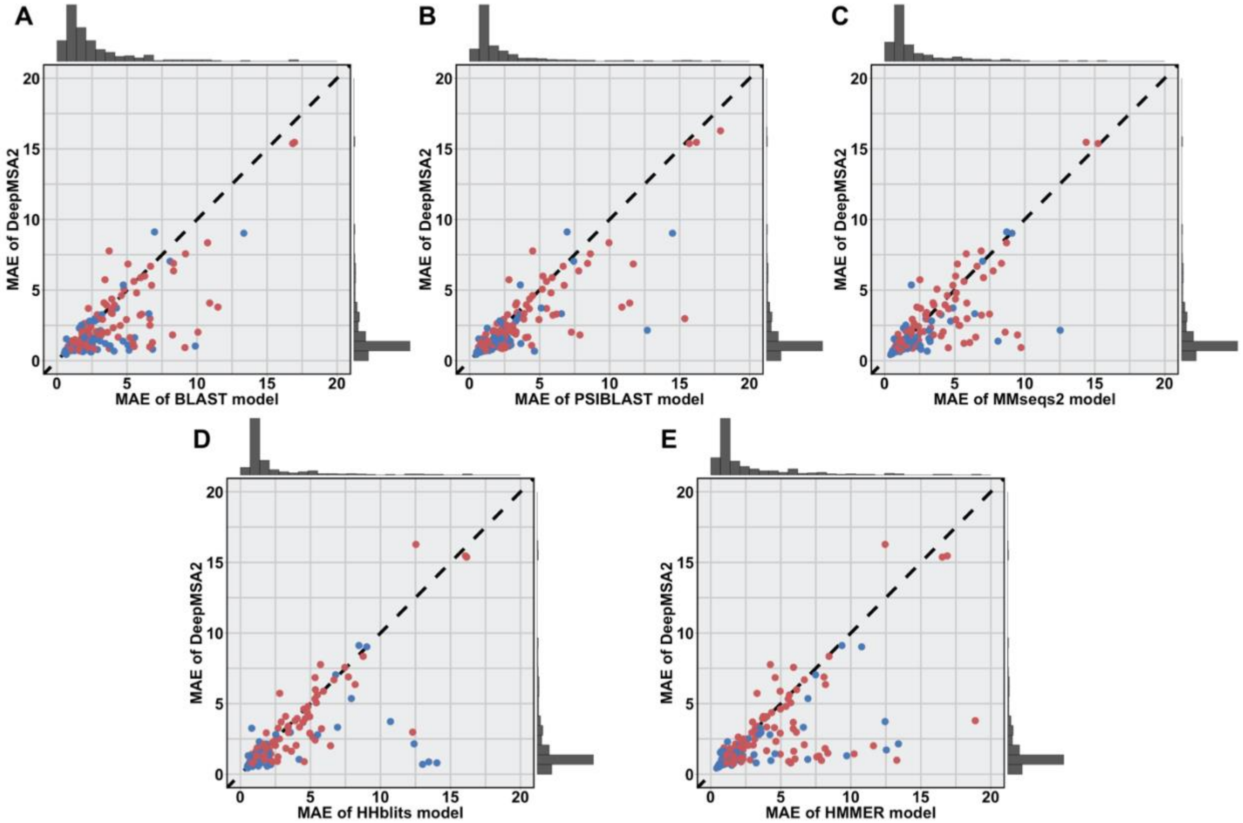

Figure S1. Head-to-head comparisons of the top 5*L* long-range mean absolute distance errors (*MAE*) between DeepMSA2 and (A) BLAST, (B) PSIBLAST, (C) MMseqs2, (D) HHblits, and (E) HMMER, calculated on 271 monomer protein domains from CASP13-15. Points below the diagonal indicate better performance by DeepMSA2 relative to each control. This analysis has excluded 22 domains from protein complexes (for example, H1137, which forms an interwound alpha-helix barrel, see **Figure 6B**), for which the contact/distance maps for each of the domains are irrelevant for DeepPotential predictions.

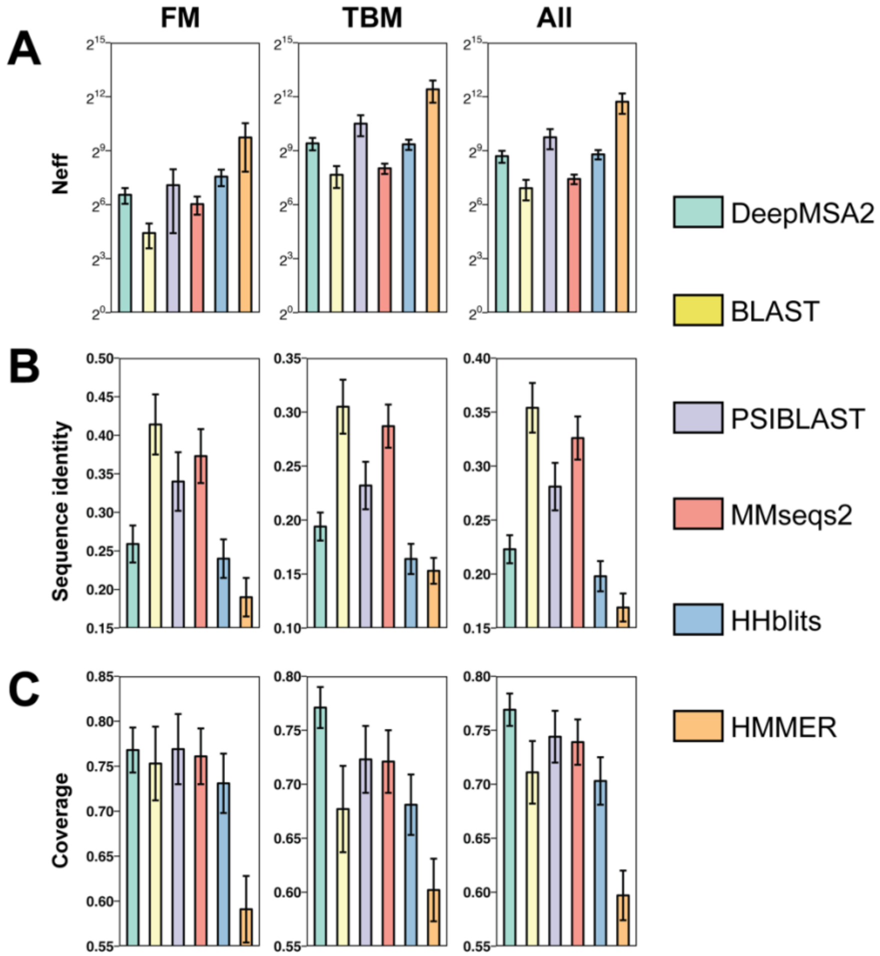

Figure S2. Comparisons of MSAs generated by DeepMSA2 and five control methods on 293 CASP monomer protein domains. (A) The number of the effective sequences (*Neff*); (B) the average sequence identity (*SeqId*) between the query and the homologous sequences in the MSAs; (C) the average alignment coverage (*cov*) between the query and the homologous sequences in the MSAs on FM, TBM, and all CASP monomer proteins. For the 'All' column, n=293 monomer domains from CASP13-15; for the 'TBM' column, n=161 template-based modeling (TBM) monomer domains from CASP13-15; for the 'FM' column, n=132 free modeling (FM) monomer domains from CASP13-15. The height of each bar indicates the mean value and error bar depicts the 95% confidence interval for each variable using Student's t-distribution.

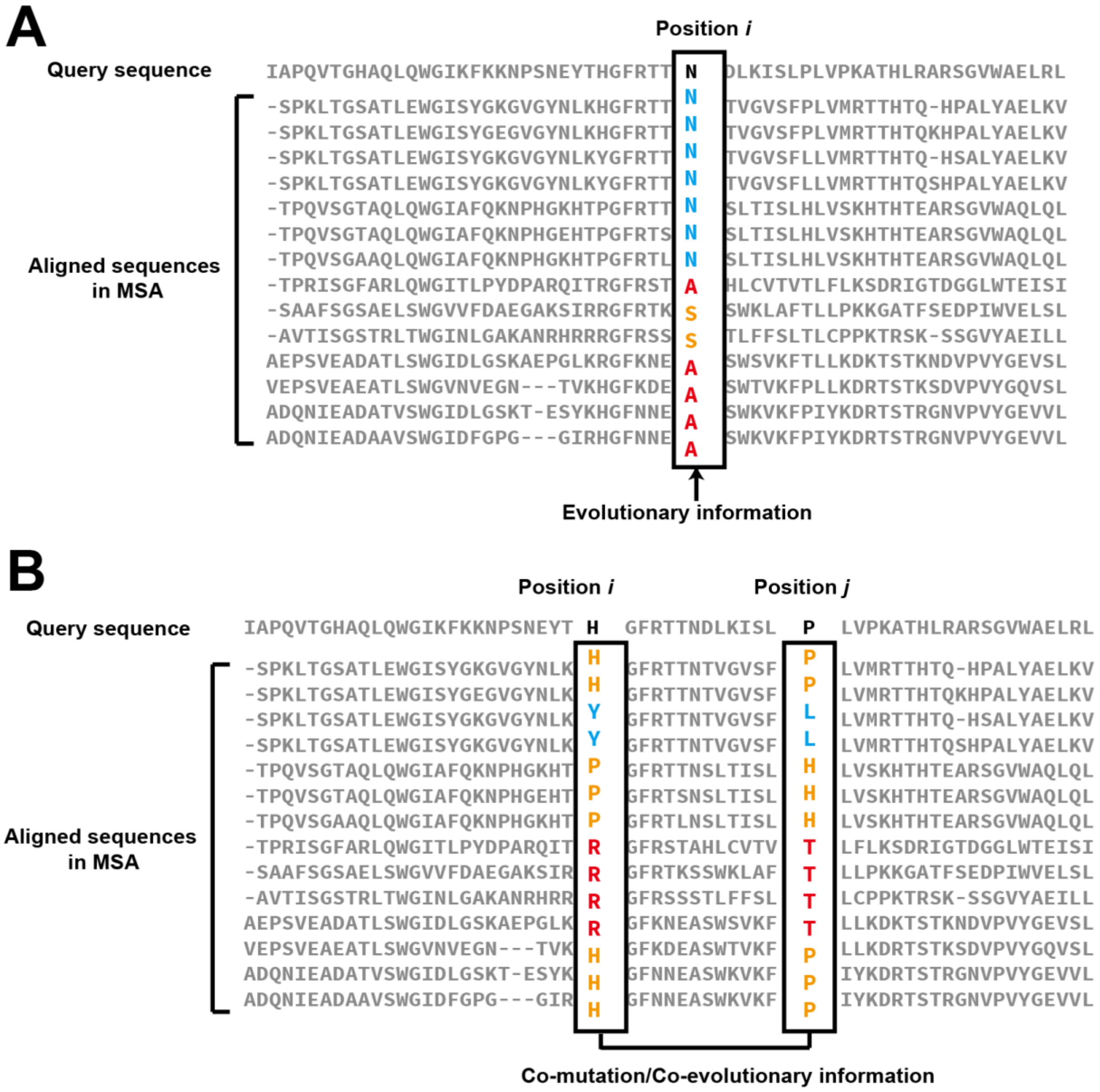

Figure S3. An illustration of (A) evolutionary information and (B) co-evolutionary information contained in multiple sequence alignments.

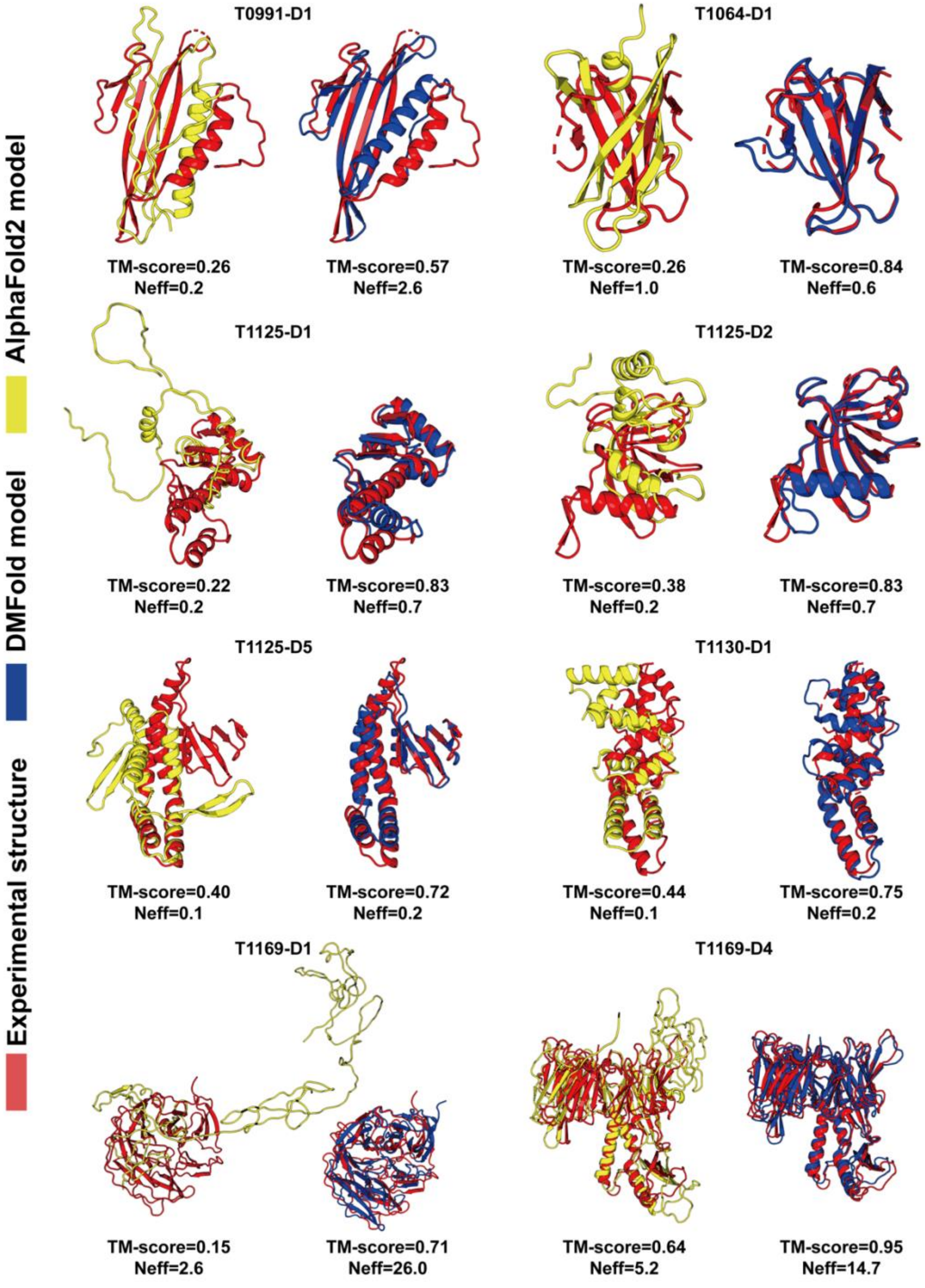

Figure S4. Case studies on domains from CASP13, CASP14, and CASP15, where the TM-scores of DMFold models (Right) have improved by more than 0.3 over those obtained using AlphaFold2 (Left).

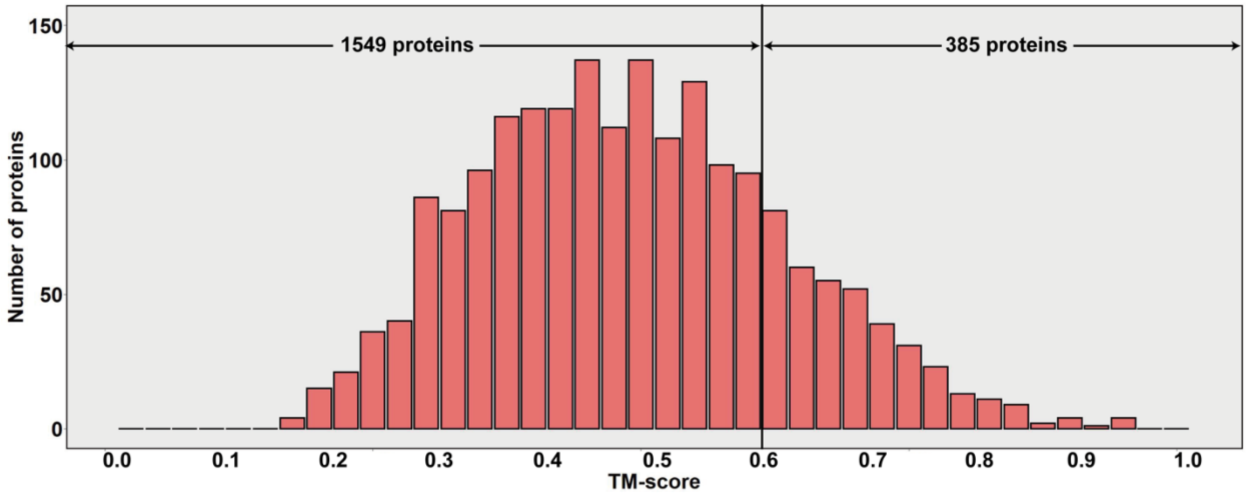

Figure S5. Structural comparisons between DMFold models and AlphaFold2 DB models on 1,934 human proteins for which the DMFold creates high-quality models with pLDDT ⩾ 0.7, while AlphaFold2 DB models have a confidence score of pLDDT<0.7. The histogram shows the distribution of TM-scores between DMFold and AlphaFold2 DB on each target. There are 385 of 1,934 targets, where two methods generate similar modes with TM-scores between two methods' models of ⩾0.6, where the rest of 1549 (80%) have TM-score <0.6.

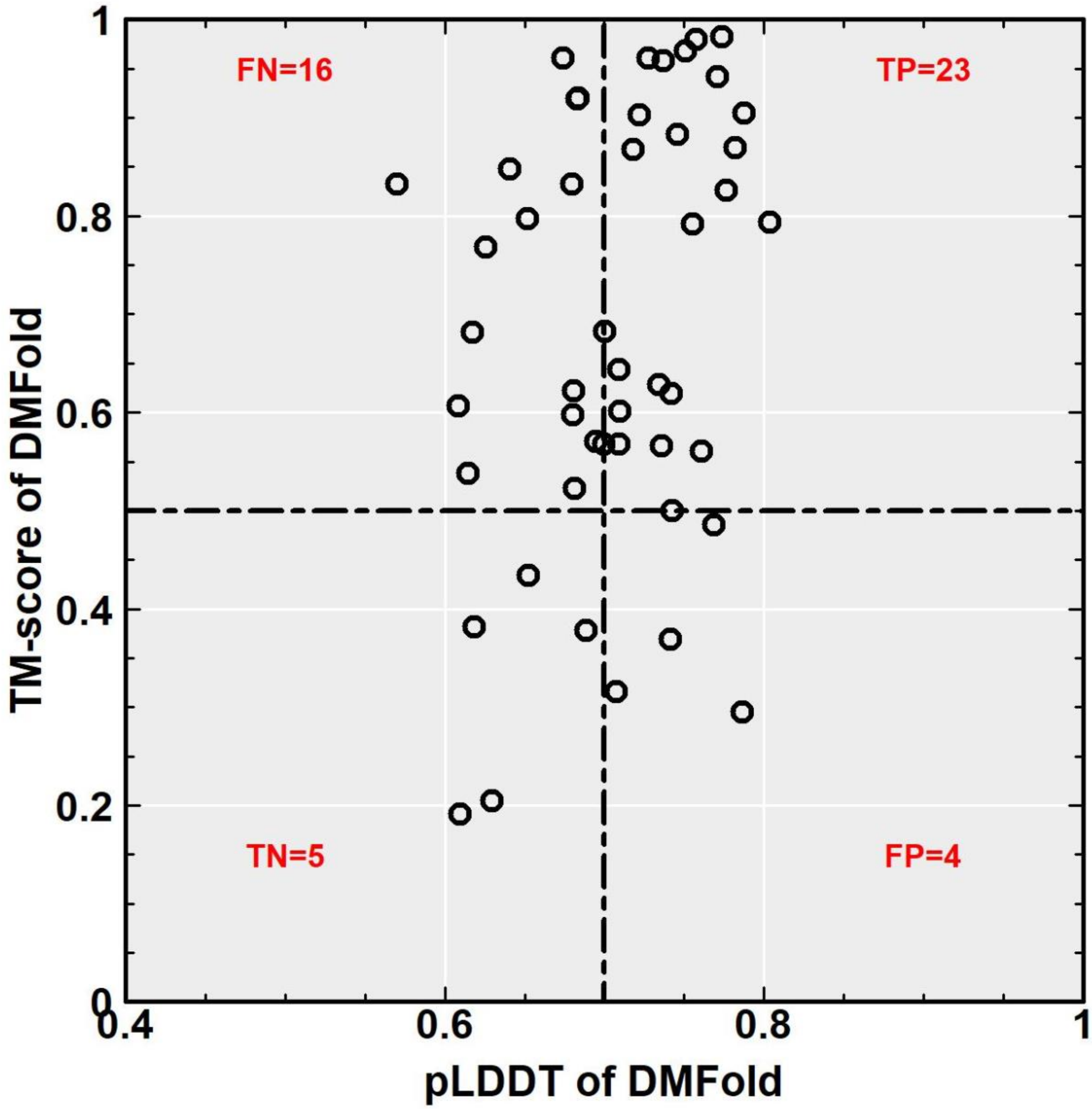

Figure S6. A head-to-head comparison between TM-score and pLDDT for final models by DMFold on 48 human proteins that have recently solved experimental structures, testing the performance of pLDDT as a binary classifier for whether a model is correctly folded (using pLDDT≥0.7 as the model-based prediction, and TM-score between the model and the experimental structure >0.5 as the ground truth). 'TP' means the number of true positive models, where DMFold models are predicted as foldable with a pLDDT ≥0.7, and are also actually foldable with a TM-score ≥0.5. 'FP' means the number of false positive models, where DMFold models are predicted as foldable with a pLDDT ≥0.7, but are actually non-foldable with a TM-score <0.5. 'TN' means the number of true negative models, where DMFold models are predicted as non-foldable with a pLDDT <0.7, and are also actually non-foldable with a TM-score <0.5. 'FN' means the number of false positive models, where DMFold models are predicted as non-foldable with a pLDDT <0.7, but are actually foldable with a TM-score ≥0.5.

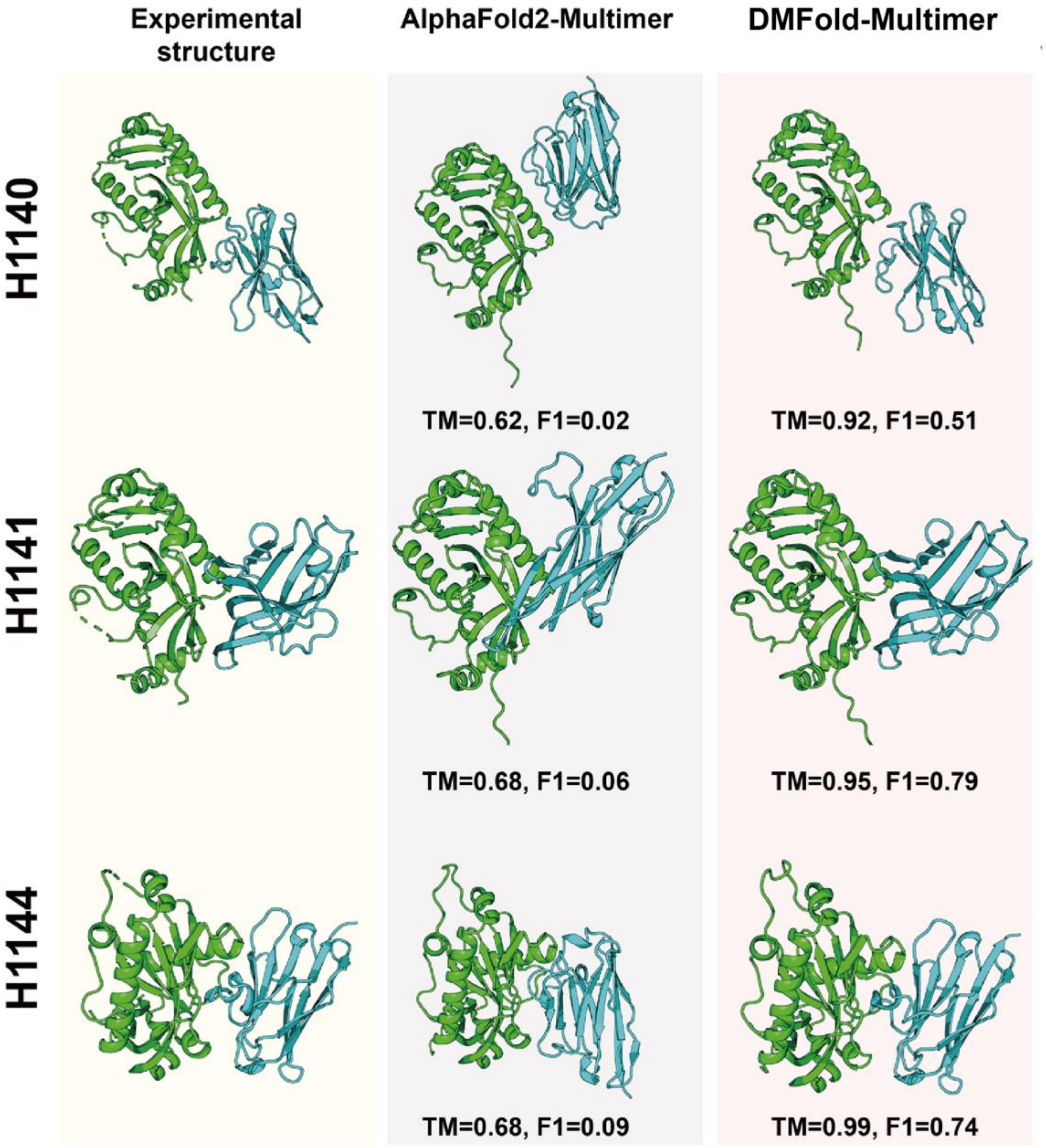

Figure S7. Illustrative examples of H1140, H1141, and H1144 in the CASP15 Multimeric Modeling Section, which are all nanobody-antigen complexes. The first column shows the experimentally solved structures, while the second and third columns are the predicted models by AlphaFold2-Multimer and DMFold-Multimer, respectively. 'TM' means TM-score of the complex models. 'F1' represents the Interface Contact Score (ICS), or F1 score, which is defined as 2*TP/(2*TP+FP+FN), where TP is the number of correctly predicted interface contacts, FP is the number of wrongly predicted interface contacts from the model which are not contacts in the experimental structure, and FN is the number of Interface contacts present in the experimental structure but predicted as non-contacts in the model.

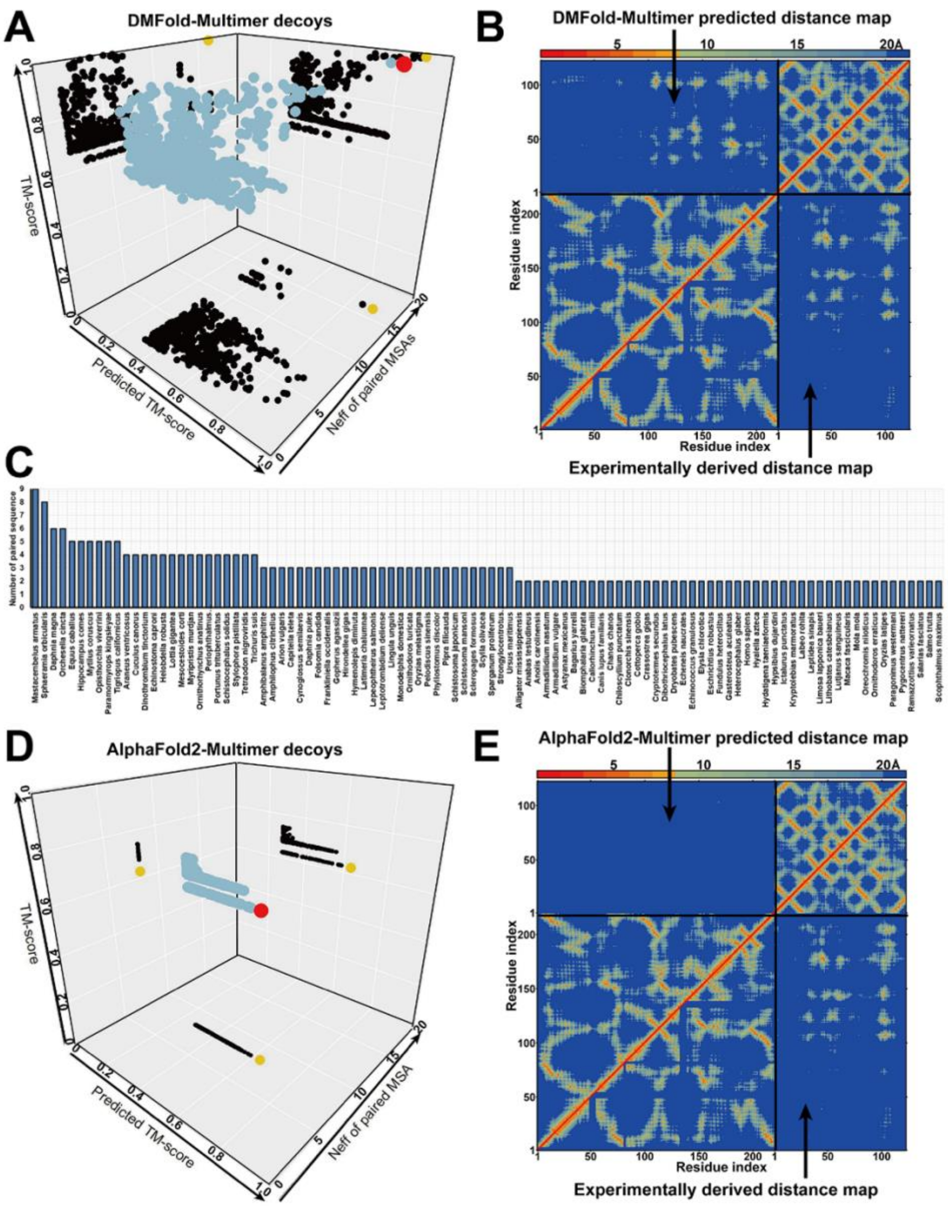

Figure S8. Case study of Target H1144 from the CASP15 Multimeric Modeling Section, which is a nanobody-antigen complex. (A) 3D scatter plot for TM-score, predicted TM-score, and *Neff* of paired MSAs on DMFold-Multimer decoys. Here, the predicted TM-score is defined by pTMS=0.2*pTM+0.8*ipTM, where pTM and ipTM are predicted TM-scores for monomer and interface models, respectively, following AlphaFold2 modeling. The larger-sized cyan points are 3D points, representing DMFold-Multimer decoys with different TM-scores, predicted TM-scores, and *Neff* of paired MSAs, where the red point refers to the 3D point corresponding to the decoy with the highest predicted TM-score. The smaller-sized black points represent the projection of 3D cyan points on the 2D planes, where the yellow points indicate the projection of the 3D red point on each of the 2D planes. Here, some DMFold-Multimer decoys have very high TM-scores as well as high predicted TM-scores, so they can be correctly selected as the final model based on the highest predicted TM-score. (B) The residue-residue distance map (heat map) for the model with the highest predicted TM-score from DMFold-Multimer (upper triangle) compared to that calculated from the experimental structure (lower triangle). (C) Top 100 species contributing to the paired MSA for H1144 ranked by the number of paired sequences. (D) Same as in panel ‘A’, but modeled with AlphaFold2-Multimer. Note that the panel ‘D’ has the same number of points (decoys) as panel ‘A’, but most of points overlap, and no high-quality models are generated. (E) Same as in panel ‘B’, but modeled with AlphaFold2-Multimer.

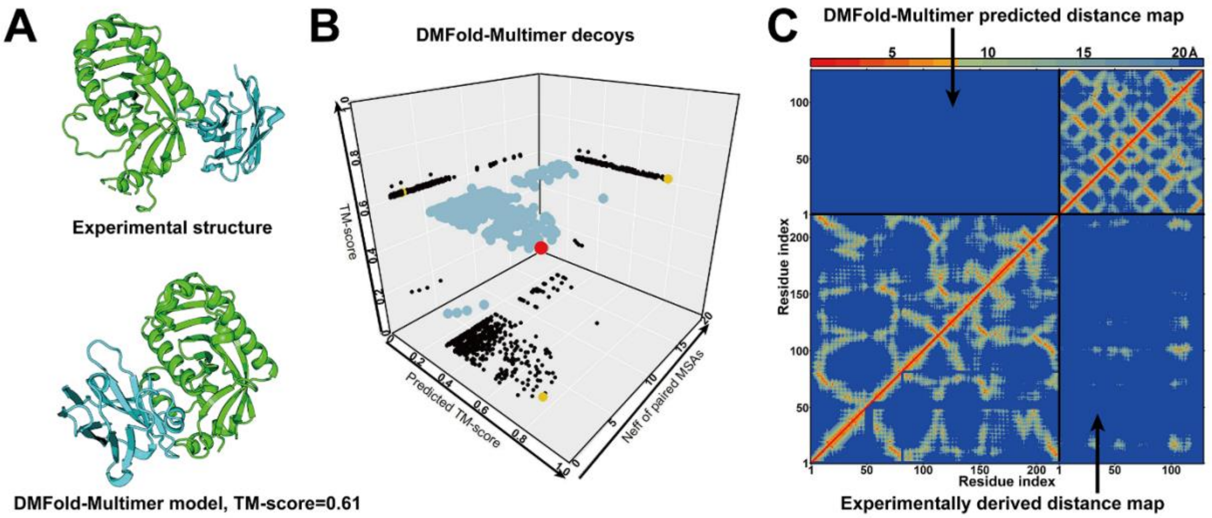

Figure S9. Case study of target H1142 from the CASP15 Multimeric Modeling Section, which is a nanobody-antigen complex. (A) The experimental structure and the DMFold-Multimer model for H1142. (B) The 3D scatter plot for TM-score, predicted TM-score, and *Neff* of paired MSAs on DMFold-Multimer decoys of H1142. The larger-sized cyan points are 3D points, representing DMFold-Multimer decoys with different TM-score, predicted TM-score, and *Neff* of paired MSAs, where the red point is the 3D point corresponding to the decoy with the highest predicted TM-score. The smaller-sized black points represent the projection of 3D cyan points on each 2D plane, where the yellow points indicate the projection of the 3D red point on each of the 2D planes. (C) The residue-residue distance map (heat map) for the model with the highest predicted TM-score from DMFold-Multimer (upper triangle) versus that calculated from the experimental structure (lower triangle) for H1142.

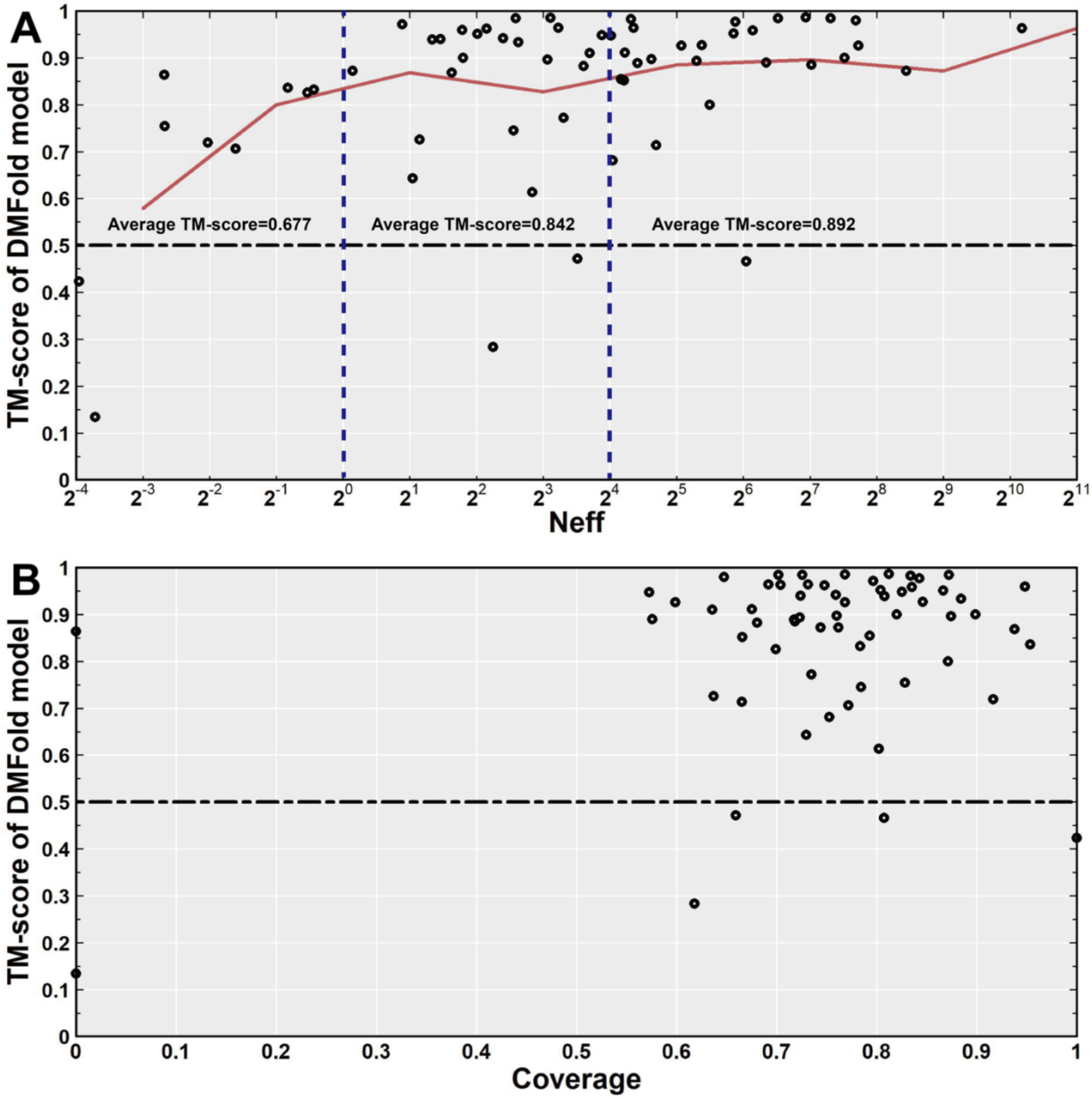

Figure S10. TM-score of DMFold models versus (A) the *Neff* of DeepMSA2 MSAs, and (B) the alignment coverage between the query and homologous sequences of the DeepMSA2 MSAs on 62 CASP13-15 ‘FM’ monomer protein domains. The ‘FM’ domains that came from protein complex are excluded in this analysis due to possible interference from binding partners. The red line indicates the average TM-score in each *Neff* bin. Two approximate thresholds, *Neff*=2^0 and *Neff*=2^4, are plotted by blue dashed lines. The average TM-scores with *Neff* lower than 2^0, between 2^0 and 2^4, and higher than 2^4 are roughly below 0.70, approximate 0.85, and higher than 0.90, respectively. If a domain does not have any homologous sequence in the MSA, we define the coverage as 0.

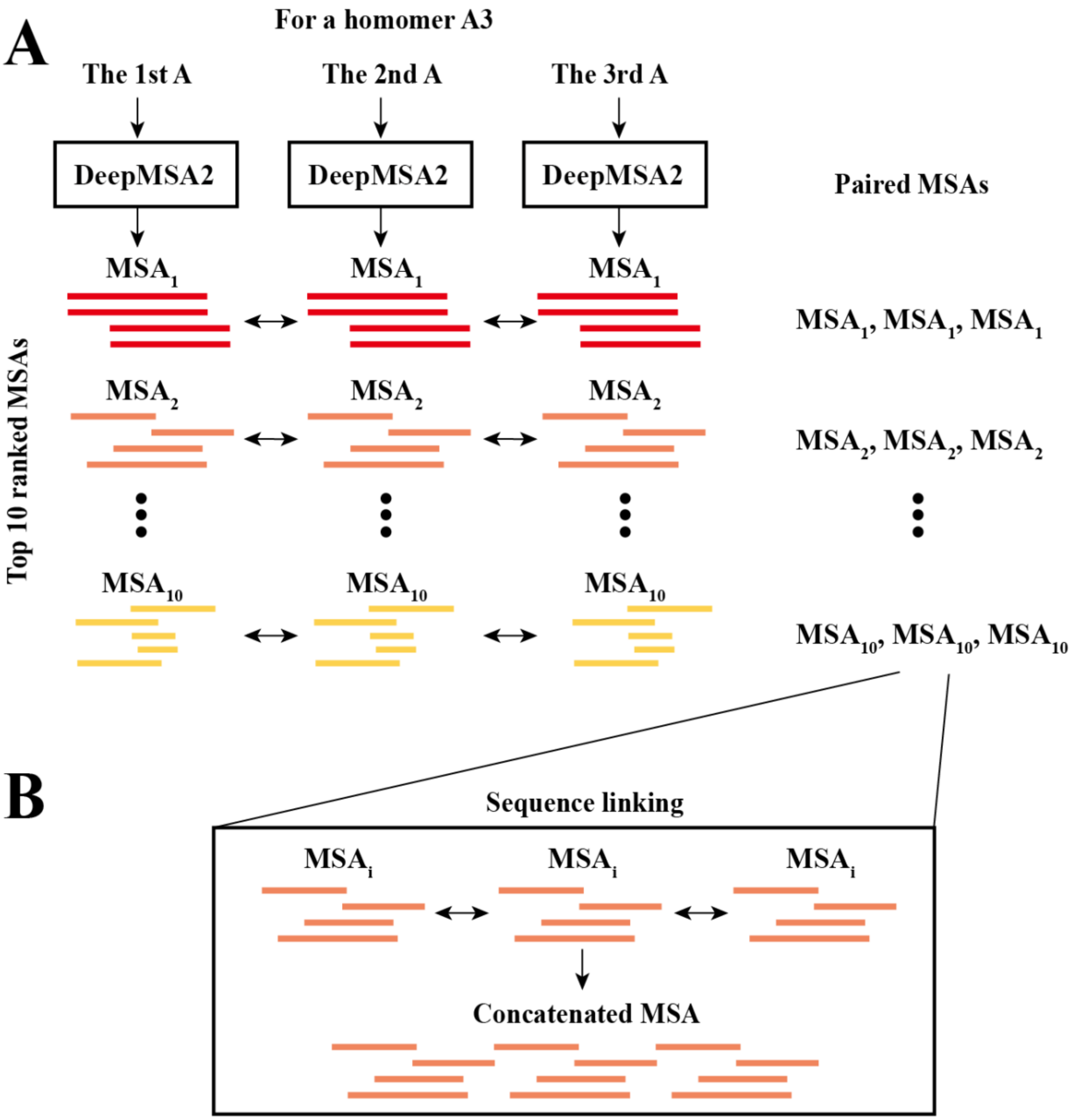

Figure S11. An illustration of the process used to generate paired MSAs in DeepMSA2-Multimer for a homomeric A3 complex (i.e., a homotrimer). (A) DeepMSA2-Monomer is used to generate a set of up to ten MSAs for the monomer protein sequence. After pLDDT score ranking, ten multimer MSAs are created by concatenating each of the monomer MSAs three times side-by-side. (B) The multimeric MSA is generated by concatenating the same monomer MSAs side-by-side an appropriate number of times.

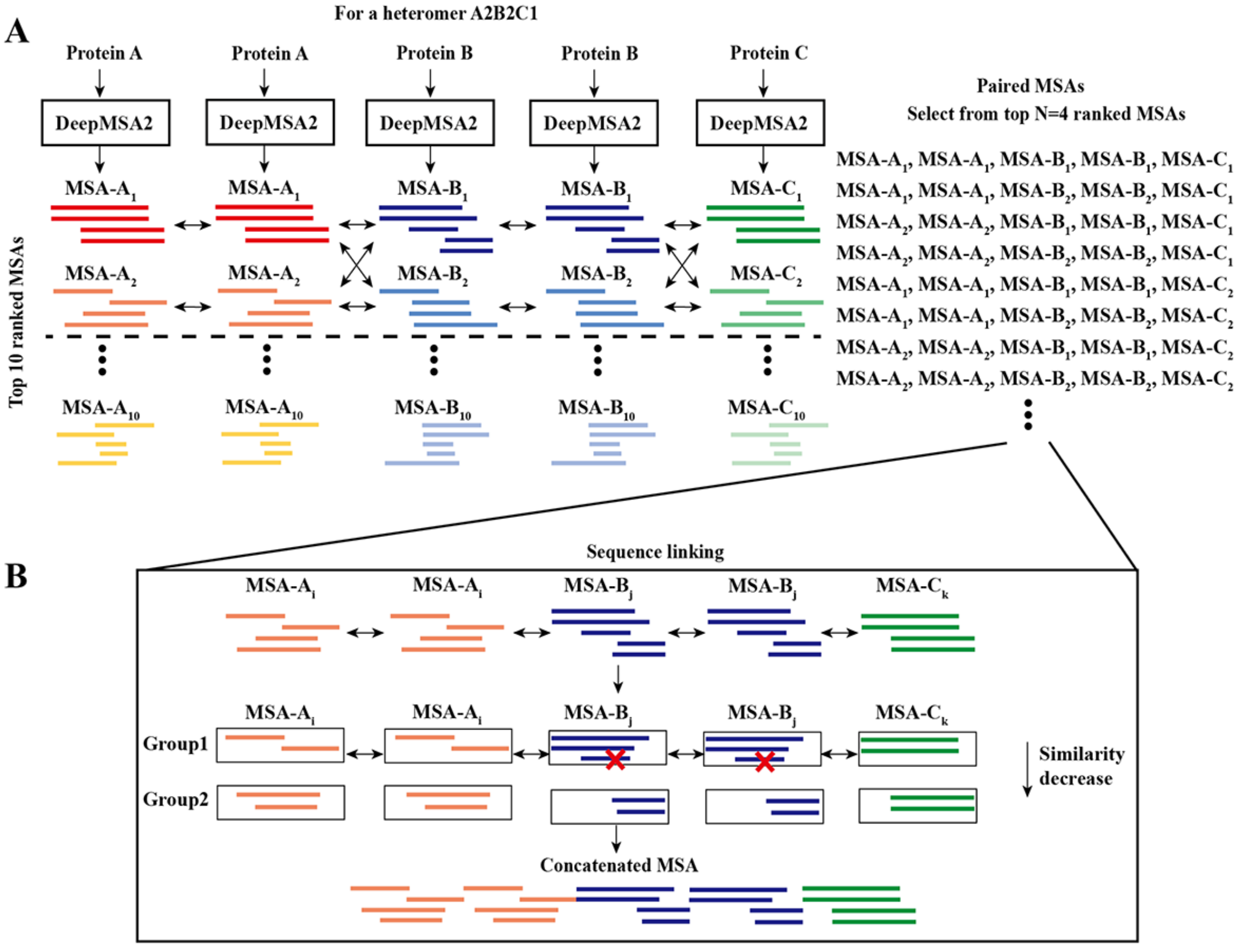

Figure S12. An illustration of the process used to generate paired MSAs in DeepMSA2-Multimer for an A2B2C1 heteromeric complex. (A) For each unique sequence chain, DeepMSA2-Monomer is first used to generate and rank up to ten candidate MSAs. Up to 64 complex MSAs are then generated by enumerating and linking all top monomer MSAs ordered by DeepMSA2-Monomer, i.e., 111, 112, 113, 114, 121, 122, 123, ... (B) The final multimeric MSAs are created by a two-step process: First, the sequences in each monomer MSA are grouped based on the UniProt annotated species and ranked based on the sequence identity to the query sequence. Second, the sequences within the same group are concatenated based on the ranking order by the minimum number of sequences from any of the component monomeric MSAs, where the red 'x' in figure means deleting the extra sequences.

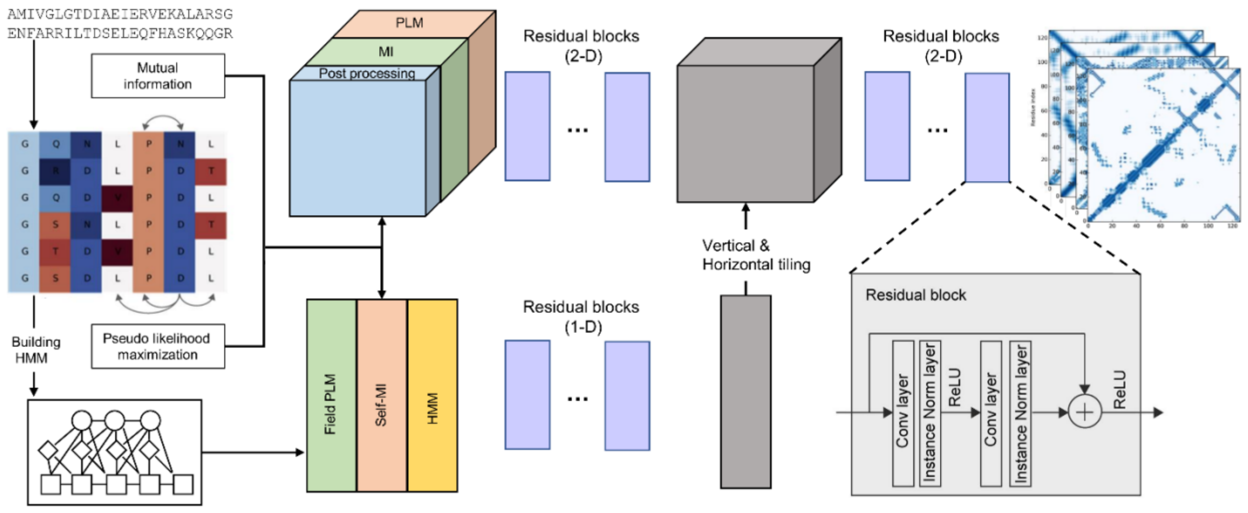

Figure S13. Architecture of DeepPotential networks for protein contact and distance map prediction (see **Methods** section in the main text and references cited therein for additional detail).

Supplementary Tables

Table S1. Benchmark results for the first threading template of HHsearch based on DeepMSA2's MSAs or the indicated third-party methods' MSAs on 287 CASP13, CASP14, and CASP15 monomer domains (six domains are excluded from all 293 CASP domains because HHsearch failed in generating results with some of the MSAs). *P*-values were calculated between TM-scores from DeepMSA2 and other third-party methods by paired one-sided Student's t-tests. The bold fonts highlight the best performance in each category.

Domain type	Method	TM-score	*P*-value
All	DeepMSA2	**0.492**	-
	BLAST	0.454	6.35E-14
	PSIBLAST	0.448	5.12E-19
	MMseqs2	0.469	2.72E-08
	HHblits	0.463	8.60E-14
	HMMER	0.448	1.09E-16
FM	DeepMSA2	**0.286**	-
	BLAST	0.245	9.41E-08
	PSIBLAST	0.247	3.41E-08
	MMseqs2	0.261	2.36E-04
	HHblits	0.264	1.16E-06
	HMMER	0.238	8.64E-10
TBM	DeepMSA2	**0.668**	-
	BLAST	0.632	7.94E-08
	PSIBLAST	0.619	5.44E-13
	MMseqs2	0.645	1.74E-05
	HHblits	0.633	5.96E-09
	HMMER	0.627	8.81E-09

Table S2. Long-range contact prediction precision by DeepPotential using MSAs from DeepMSA2 and the five control methods on CASP13, CASP14, and CASP15 monomer protein domains. Here, the long-range contacts are defined as contacts (i.e., distance<8Å) with sequence separation $|i\text{-}j|\geq 24$. *P*-values were calculated between the results from DeepPotential using DeepMSA2 MSA and third-party methods by paired one-sided Student's t-tests. The analysis has excluded the 22 domains from all 293 CASP domains because (i) DeepPotential failed in generating results for some of the MSAs (for example, HMMER MSA that contains too many sequences), or (ii) the target is from the hard protein complexes for DeepPotential to make a prediction (for example, domains from a protein complex H1137, which are interwind helices with few long-range contacts in individual chains). The bold fonts highlight the best performance in each category.

**Domain Type**	**Method**	**Long range**					
		L/5	***P*-value**	***L/2***	***P*-value**	***L***	***P*-value**
All	DeepMSA2	**0.831**	-	**0.737**	-	**0.601**	-
	BLAST	0.734	2.06E-13	0.635	2.06E-18	0.514	1.61E-21
	PSIBLAST	0.790	5.47E-06	0.698	5.71E-07	0.566	3.99E-09
	MMseqs2	0.790	4.14E-06	0.699	4.66E-06	0.568	6.57E-07
	HHblits	0.788	1.23E-05	0.694	4.22E-06	0.559	1.23E-07
	HMMER	0.740	1.37E-10	0.655	1.53E-11	0.538	4.57E-12
FM	DeepMSA2	**0.721**	-	**0.608**	-	**0.473**	-
	BLAST	0.592	4.53E-09	0.487	2.17E-10	0.376	2.28E-11
	PSIBLAST	0.656	5.02E-05	0.552	1.48E-05	0.424	4.33E-07
	MMseqs2	0.653	5.83E-05	0.554	1.74E-04	0.428	5.63E-05
	HHblits	0.676	1.14E-03	0.576	5.44E-03	0.451	1.26E-02
	HMMER	0.576	8.77E-09	0.485	1.88E-09	0.381	2.37E-09
TBM	DeepMSA2	**0.926**	-	**0.849**	-	**0.711**	-
	BLAST	0.855	4.29E-06	0.763	7.57E-10	0.631	6.37E-12
	PSIBLAST	0.904	1.63E-02	0.823	4.55E-03	0.688	8.46E-04
	MMseqs2	0.908	1.10E-02	0.824	4.45E-03	0.689	1.79E-03
	HHblits	0.883	1.77E-03	0.794	1.33E-04	0.652	1.16E-06
	HMMER	0.881	1.21E-03	0.801	4.26E-04	0.672	1.29E-04

Table S3. Top 5*L* long-range mean absolute distance error (*MAE*) on CASP13, CASP14, and CASP15 monomer protein domains. The analysis has excluded the 22 domains from all 293 CASP domains because (i) DeepPotential failed in generating results for some of the MSAs (for example, HMMER MSA that contains too many sequences), or (ii) the target is from the hard protein complexes for DeepPotential to make a prediction (for example, domains from a protein complex H1137, which are interwind helices with few long-range contacts in individual chains).The residue-residue distances were predicted by DeepPotential with MSAs from DeepMSA2 and the five control methods. *P*-values were calculated between *MAE*s from DeepMSA2 and other five control methods by paired one-sided Student's t-tests. The bold fonts highlight the best performance in each category.

**Domain Type**	**Method**	***MAE***	***P*-value**
All	DeepMSA2	**2.22**	**-**
	BLAST	3.09	1.62E-22
	PSIBLAST	2.70	5.79E-12
	MMseqs2	2.68	1.03E-07
	HHblits	2.74	1.26E-09
	HMMER	2.98	4.80E-13
FM	DeepMSA2	**3.42**	**-**
	BLAST	4.44	1.55E-10
	PSIBLAST	4.11	2.16E-07
	MMseqs2	4.07	3.92E-04
	HHblits	3.74	1.31E-04
	HMMER	4.49	8.83E-08
TBM	DeepMSA2	**1.20**	**-**
	BLAST	1.94	3.88E-15
	PSIBLAST	1.49	8.83E-07
	MMseqs2	1.48	1.37E-05
	HHblits	1.89	6.70E-07
	HMMER	1.69	2.08E-07

Table S4. Summary of the MSAs produced by DeepMSA2, BLAST, PSIBLAST, MMseqs2, HHblits, and HMMER on 293 CASP13, CASP14, and CASP15 monomer proteins. '*Nseq*' is the number of the sequences in the MSA, and '*Neff*' is the number of effective sequences in the MSA; the mean across each set of considered cases is shown. *P*-values were calculated between measures from DeepMSA2 and the control methods using paired one-sided Student's t-tests.

Domain Type	Method	*Nseq*	*P*-value	*Neff*	*P*-value	Sequence Identity	*P*-value	Coverage	*P*-value
All	DeepMSA2	8642.2	-	415.3	-	0.223	-	0.770	-
	BLAST	3795.4	1.40E-25	120.9	2.12E-32	0.354	9.99E-01	0.712	4.15E-03
	PSIBLAST	50243.8	9.99E-01	858.4	9.92E-01	0.281	9.99E-01	0.744	3.85E-01
	MMseqs2	3367.9	3.34E-14	172.4	1.44E-15	0.326	9.99E-01	0.739	3.46E-03
	HHblits	9309.4	9.78E-01	444.9	8.86E-01	0.198	2.57E-13	0.703	1.10E-19
	HMMER	100649.8	9.99E-01	3377.3	9.99E-01	0.169	7.64E-21	0.597	3.88E-39
FM	DeepMSA2	2279.3	-	93.7	-	0.259	-	0.768	-
	BLAST	1082.9	4.03E-17	21.5	7.90E-18	0.414	9.99E-01	0.753	8.52E-01
	PSIBLAST	9059.9	9.99E-01	135.8	9.74E-05	0.340	9.99E-01	0.769	9.90E-01
	MMseqs2	1497.4	2.36E-04	65.5	5.43E-06	0.374	9.99E-01	0.761	2.76E-01
	HHblits	4318.0	9.44E-01	188.7	9.72E-01	0.240	1.77E-03	0.731	2.14E-04
	HMMER	27459.9	9.99E-01	851.7	9.99E-01	0.190	1.20E-09	0.591	3.34E-17
TBM	DeepMSA2	13859.0	-	678.9	-	0.194	-	0.771	-
	BLAST	6019.2	5.91E-13	202.4	6.21E-18	0.305	9.99E-01	0.677	1.24E-05
	PSIBLAST	84009.4	9.99E-01	1450.9	9.99E-01	0.232	9.99E-01	0.723	7.86E-03
	MMseqs2	4901.5	6.29E-12	260.0	5.81E-12	0.287	9.99E-01	0.721	1.10E-03
	HHblits	13401.7	7.58E-01	655.0	4.63E-01	0.164	5.38E-13	0.681	6.94E-18
	HMMER	160656.4	9.99E-01	5448.0	9.99E-01	0.153	1.01E-13	0.602	5.48E-24

Table S5. The average values of the number of effective sequences (*Neff*), number of homologous sequences (N_{seq}), and TM-scores of final models by three different programs on 132 FM domains in CASP13, CASP14 and CASP15. 'DMFold-noh' refers to the program using MSAs from DeepMSA2 but without using the three in-house metagenome databases (TaraDB, MetaSourceDB, and JGIclust). *P*-values were calculated between TM-scores of DMFold and other programs using paired one-sided Student's t-tests.

**Method**	***Neff***	N_{seq}	**TM-score**	***P*-value**
DMFold	93.7	2279	0.8207	-
AlphaFold2	84.5	2724	0.7807	1.82E-04
DMFold-noh	85.3	2160	0.8034	1.65E-05

Table S6. The average TM-score of final models produced by DMFold and AlphaFold2 DB on 48 human proteome proteins that have low AlphaFold2 DB pLDDT scores and recently solved experimental structures. *P*-value was calculated between TM-scores of DMFold and AlphaFold2 using a paired one-sided Student's t-test.

**Method**	**TM-score**	***P*-value**
DMFold	0.679	-
AlphaFold2 DB	0.630	1.46E-04

Table S7. The set of protein structures comprising our protein complex dataset, including 14 heteromer complexes and 40 homomer complexes from CASP13 and CASP14.

Target	Stoichiometry	CASP	Target	Stoichiometry	CASP
H0953	A3B1	CASP13	T0991o	A2	CASP13
H0957	A1B1	CASP13	T0995o	A8	CASP13
H0968	A2B2	CASP13	T0997o	A2	CASP13
H0974	A1B1	CASP13	T0998o	A2	CASP13
H0980	A2B2	CASP13	T1000o	A2	CASP13
H0986	A1B1	CASP13	T1001o	A2	CASP13
H1015	A1B1	CASP13	T1003o	A2	CASP13
H1017	A1B1	CASP13	T1004o	A3	CASP13
H1019	A1B1	CASP13	T1006o	A2	CASP13
H1045	A1B1	CASP14	T1009o	A2	CASP13
H1047	A1B1	CASP14	T1010o	A2	CASP13
H1065	A1B1	CASP14	T1016o	A2	CASP13
H1072	A2B2	CASP14	T1018o	A2	CASP13
H1097	A1B1C1D1E1	CASP14	T1020o	A3	CASP13
T0960o	A3	CASP13	T1032o	A2	CASP14
T0961o	A4	CASP13	T1034o	A4	CASP14
T0963o	A3	CASP13	T1038o	A2	CASP14
T0966o	A2	CASP13	T1048o	A4	CASP14
T0970o	A2	CASP13	T1054o	A2	CASP14
T0973o	A2	CASP13	T1061o	A3	CASP14
T0976o	A2	CASP13	T1062o	A3	CASP14
T0977o	A3	CASP13	T1070o	A3	CASP14
T0979o	A3	CASP13	T1073o	A4	CASP14
T0981o	A3	CASP13	T1080o	A3	CASP14
T0984o	A2	CASP13	T1083o	A2	CASP14
T0988o	A3	CASP13	T1084o	A2	CASP14
T0989o	A3	CASP13	T1087o	A2	CASP14

Table S8. The structure prediction ability of DMFold-Multimer and AlphaFold2-Multimer on 14 heteromer and 40 homomer complex targets collected from CASP13 and CASP14 (see main text for details). *P*-values were calculated between TM-scores for DMFold-Multimer and AlphaFold2-Multimer models using paired one-sided Student's t-tests. #{TM≥0.5} is the number of targets with a TM-score ≥0.5.

Method	Target Type	TM-score	*P*-value	#{TM≥0.5}
**DMFold-Multimer**	**All**	0.8344	-	50
	**Heteromer**	0.9295	-	14
	**Homomer**	0.8010	-	36
**AlphaFold2-Multimer**	**All**	0.7434	2.44E-04	45
	**Heteromer**	0.8953	3.92E-02	14
	**Homomer**	0.6902	1.20E-03	31

Table S9. Summary of the protein complex modeling results for all 87 participant groups in the CASP15 experiment. The ranking of the groups is based on the sum of Z-score with threshold >0.0 with data taken from the official CASP15 website (https://predictioncenter.org/casp15/zscores_multimer.cgi). Here, DMFold-Multimer was registered as 'Zheng', and the standard version AlphaFold2-Multimer (operated by the Elofsson lab) was registered as 'NBIS-AF2-multimer'. Following the CASP Assessors' formula, Z-score=Z-score (ICS) + Z-score (IPS) + Z-score (LDDT) + Z-score (TM-score). TM-score and LDDT score are measures that are used for qualifying the global fold of the model, and Interface Contact Score (ICS) and Interface Patch Score (IPS) are scoring function that are used to evaluate the interface of the model. ICS is the F1 score calculated from the contacts derived from model and experimental structures, respectively, and IPS is the Jaccard index of the contact residues derived from model and experimental structures.

Ranking	Group Names	Sum Z-score (>0.0)	Ranking	Group Names	Sum Z-score (>0.0)
1	Zheng (DMFold-Multimer)	35.30	45	GinobiFold-SER	6.78
2	Venclovas	29.15	46	GuijunLab-DeepDA	6.45
3	Wallner	28.14	47	TRFold	6.13
4	Yang-Multimer	24.69	48	Zou	6.12
5	Yang	24.17	49	GinobiFold	5.55
6	Kiharalab	21.82	50	Manifold-X	5.44
7	MULTICOM_human	20.72	51	WL_team	5.31
8	Manifold	20.29	52	DELCLAB	5.27
9	McGuffin	19.89	53	Agemo_mix	5.12
10	MULTICOM	19.65	54	Kozakov-Vajda	5.02
11	Manifold-E	18.86	55	UNRES	4.89
12	MULTICOM_qa	18.35	56	Dfolding	4.85
13	PEZYFoldings	17.95	57	ShanghaiTech-TS-SER	4.72
14	Dfolding-server	17.01	58	FoldEver-Hybrid	4.64
15	MULTICOM_deep	16.29	59	FoldEver	4.32
16	CoDock	16.26	60	Manifold-LC-E	4.05
17	BAKER	15.91	61	bio3d	3.84
18	UltraFold	15.78	62	Fernandez-Recio	3.82
19	BeijingAIProtein	15.72	63	ChaePred	3.47
20	UltraFold_Server	15.71	64	OpenFold	2.83
21	Elofsson	15.69	65	OpenFold-SingleSeq	2.83
22	Takeda-Shitaka_Lab	15.63	66	Aichemy_LIG3	2.74
23	MultiFOLD	15.24	67	Aichemy_LIG	2.71
24	MUFold_H	15.10	68	Aichemy_LIG2	2.71
25	colabfold_human	14.34	69	ddquest	2.27
26	MUFold	14.09	70	KORP-PL	2.19
27	Pierce	14.04	71	Convex-PL-R	2.03
28	Kiharalab_Server	13.52	72	zax	2.00
29	ColabFold	12.77	73	UTMB	1.92
**30**	**NBIS-AF2-multimer**	**12.27**	74	Convex-PL	1.72
31	RaptorX-Multimer	11.92	75	TB_model_prediction	1.31
32	Grudinin	11.89	76	XRC_VU	1.15
33	DMP	11.42	77	Graphen_Medical	0.94
34	Yang-Server	10.50	78	ESM-single-sequence	0.89
35	SHT	10.20	79	Gonglab-THU	0.53
36	Dfolding-refine	9.33	80	Cerebra	0.53
37	ShanghaiTech	9.28	81	noxelis	0.41
38	ClusPro	9.04	82	TensorLab	0.36
39	GuijunLab-Human	8.86	83	Panlab	0.35
40	Coqualia	8.66	84	GuijunLab-Meta	0.34
41	GuijunLab-Assembly	8.60	85	FALCON2	0.00
42	FTBiot0119	7.67	86	FALCON0	0.00
43	Shen-CAPRI	7.59	87	wuqi	0.00
44	trComplex	7.44			

Table S10. Comparison of the TM-score, LDDT, Interface Contact Score (ICS), and Interface Patch Score (IPS) results between DMFold-Multimer and AlphaFold2-Multimer in the CASP15 experiment. Here, DMFold-Multimer was registered as ‘Zheng’, and the standard version AlphaFold2-Multimer (operated by the Elofsson lab) was registered as ‘NBIS-AF2-multimer’. TM-score and LDDT score are measures that are used for qualifying the global fold of the model, and Interface Contact Score (ICS) and Interface Patch Score (IPS) are scoring function that are used to qualify the interface of the model. ICS is the F1 score calculated from the contacts derived from model and experimental structures, and IPS is the Jaccard index of the contact residues derived from the model and experimental structures. *P*-values were calculated between methods by DMFold-Multimer and AlphaFold2-Multimer using paired one-sided Student’s t-tests.

**Method**	**TM-score**	***P*-value**	**LDDT**	***P*-value**	**ICS**	***P*-value**	**IPS**	***P*-value**
DMFold-Multimer	0.830	-	0.789	-	0.598	-	0.641	-
AlphaFold2-Multimer	0.719	8.23E-04	0.719	3.07E-03	0.469	2.23E-04	0.538	1.86E-04

Table S11. The monomer protein dataset used in our benchmark tests, including 48 free modeling (FM) domains and 64 template-based modeling (TBM) domains from CASP13, 37 FM domains and 50 TBM domains from CASP14, as well as 47 FM domains and 47 TBM domains from CASP15.

CASP	Domain Type	Domains
CASP13	TBM	T0954-D1,T0957s1-D2,T0959-D1,T0960-D3,T0960-D5,T0961-D1,T0963-D3, T0963-D5,T0964-D1,T0965-D1,T0966-D1,T0973-D1,T0974s1-D1,T0976-D1, T0976-D2,T0977-D1,T0977-D2,T0979-D1,T0981-D1,T0981-D4,T0981-D5, T0983-D1,T0984-D1,T0984-D2,T0985-D1,T0993s1-D1,T0993s2-D1,T0995-D1, T0996-D1,T0996-D2,T0996-D3,T0996-D4,T0996-D5,T0996-D6,T0999-D1, T0999-D2,T0999-D3,T0999-D4,T0999-D5,T1000-D1,T1002-D1,T1002-D2, T1002-D3,T1003-D1,T1004-D1,T1004-D2,T1004-D3,T1006-D1,T1009-D1, T1011-D1,T1011-D2,T1013-D1,T1014-D1,T1014-D2,T1015s2-D1,T1016-D1, T1017s1-D1,T1018-D1,T1019s2-D1,T1020-D1,T1021s1-D1,T1021s2-D1, T1022s1-D2,T1022s2-D1
	FM	T0949-D1,T0953s1-D1,T0953s2-D1,T0953s2-D2,T0953s2-D3,T0955-D1, T0957s1-D1,T0957s2-D1,T0958-D1,T0960-D1,T0960-D2,T0960-D4,T0963-D1, T0963-D2,T0963-D4,T0968s1-D1,T0968s2-D1,T0969-D1,T0970-D1,T0975-D1, T0980s1-D1,T0980s2-D1,T0981-D2,T0981-D3,T0986s1-D1,T0986s2-D1,T0987-D1, T0987-D2,T0989-D1,T0989-D2,T0990-D1,T0990-D2,T0990-D3,T0991-D1, T0992-D1,T0997-D1,T0998-D1,T1000-D2,T1001-D1,T1005-D1,T1008-D1, T1010-D1,T1015s1-D1,T1017s2-D1,T1019s1-D1,T1021s3-D1,T1021s3-D2, T1022s1-D1
CASP14	TBM	T1024-D1,T1024-D2,T1026-D1,T1030-D1,T1030-D2,T1032-D1,T1034-D1, T1045s2-D1,T1046s2-D1,T1047s2-D2,T1050-D1,T1050-D2,T1050-D3,T1052-D1, T1052-D2,T1054-D1,T1056-D1,T1057-D1,T1058-D2,T1060s2-D1,T1060s3-D1, T1061-D3,T1065s1-D1,T1067-D1,T1068-D1,T1070-D2,T1070-D3,T1070-D4, T1073-D1,T1076-D1,T1078-D1,T1079-D1,T1083-D1,T1084-D1,T1087-D1, T1089-D1,T1091-D1,T1091-D2,T1091-D3,T1091-D4,T1092-D1,T1092-D2, T1093-D2,T1094-D1,T1095-D1,T1099-D1,T1100-D1,T1100-D2,T1101-D1, T1101-D2
	FM	T1027-D1,T1029-D1,T1031-D1,T1033-D1,T1035-D1,T1037-D1,T1038-D1, T1038-D2,T1039-D1,T1040-D1,T1041-D1,T1042-D1,T1043-D1,T1046s1-D1, T1047s1-D1,T1047s2-D1,T1047s2-D3,T1049-D1,T1052-D3,T1053-D1,T1053-D2, T1055-D1,T1058-D1,T1061-D1,T1061-D2,T1064-D1,T1065s2-D1,T1070-D1, T1074-D1,T1080-D1,T1082-D1,T1090-D1,T1093-D1,T1093-D3,T1094-D2, T1096-D1,T1096-D2
CASP15	TBM	T1106s2-D1, T1109-D1, T1110-D1, T1114s2-D1, T1114s3-D1, T1119-D1, T1121-D2, T1124-D1, T1125-D3, T1127-D1, T1132-D1, T1133-D1, T1134s1-D1, T1137s1-D1, T1137s2-D1, T1137s3-D1, T1137s4-D1, T1137s5-D1, T1137s6-D1, T1137s7-D1, T1137s8-D1, T1137s9-D1, T1139-D1, T1145-D1, T1146-D1, T1147-D1, T1152-D1, T1153-D1, T1157s1-D1, T1157s2-D1, T1157s2-D2, T1157s2-D3, T1158-D1, T1158-D2, T1160-D1, T1161-D1, T1162-D1, T1163-D1, T1169-D3, T1170-D1, T1170-D2, T1173-D1, T1174-D2, T1175-D1, T1180-D1, T1183-D1, T1188-D1
	FM	T1104-D1, T1106s1-D1, T1112-D1, T1113-D1, T1114s1-D1, T1120-D1, T1120-D2, T1121-D1, T1122-D1, T1123-D1, T1125-D1, T1125-D2, T1125-D4, T1125-D5, T1125-D6, T1129s2-D1, T1130-D1, T1131-D1, T1134s2-D1, T1137s1-D2, T1137s2-D2, T1137s3-D2, T1137s4-D2, T1137s4-D3, T1137s5-D2, T1137s6-D2, T1145-D2, T1150-D1, T1151s2-D1, T1154-D1, T1154-D2, T1155-D1, T1159-D1, T1169-D1, T1169-D2, T1169-D4, T1173-D2, T1174-D1, T1177-D1, T1178-D1, T1179-D1, T1181-D1, T1181-D2, T1182-D1, T1184-D1, T1187-D1, T1194-D1

Table S12. Summary of genomic and metagenomics databases used in DeepMSA2.

Program	Database	Database Type	Sequence Type	Number of Sequences
dMSA	Uniclust30	Hidden Markov Model database	Genome	124 million
dMSA/qMSA	Uniref90	Sequence database	Genome	109 million
dMSA	Metaclust	Sequence database	Metagenome	712 million
qMSA	UniRef30	Hidden Markov Model database	Genome	231 million
qMSA	BFD	Hidden Markov Model database	Metagenome	2.2 billion
qMSA	Mgnify	Sequence database	Metagenome	305 million
mMSA	TaraDB	Sequence database	Metagenome	121 million
mMSA	MetaSourceDB	Sequence database	Metagenome	19.1 billion
mMSA	JGIclust	Sequence database	Metagenome	16.4 billion

Table S13. Impacts of BLAST filtering on the MSA generation step of the dMSA pipeline. We ran DMFold using only the dMSA stage, either with or without the BLAST filter, on 87 monomer protein domains from CASP14. *P*-values were calculated between TM-scores by DMFold utilizing dMSA with or without BLAST filter using paired one-sided Student's t-tests. #{TM≥0.5} is the number of domains with a TM-score ≥0.5.

Method	Domain Type	TM-score	*P*-value	#{TM≥0.5}
dMSA with BLAST filter	All	0.8632	-	83
	TBM	0.9137	-	50
	FM	0.7949	-	33
dMSA without BLAST filter	All	0.8545	4.67E-02	82
	TBM	0.9043	3.78E-02	49
	FM	0.7872	1.99E-01	33

Supplement Texts

Text S1. DeepMSA2 provides balanced MSAs for monomer fold-recognition and spatial restraint prediction

We chose five methodologies for generating multiple sequence alignments (MSAs), for use as control approaches compared with DeepMSA2. These include BLAST[1], HHblits[2], HMMER[3], MMseqs2[4], and PSIBLAST[5]. The commonly used approach employed by BLAST and PSIBLAST for MSA generation primarily involves searching the "NR" genomic sequence database[5] sourced from NCBI. Conversely, HHblits typically relies on either the Uniclust30 or UniRef30 genome sequence database to construct the MSA[6]. However, it should be noted that this exclusive reliance on genome databases during the MSA generation process disregards the wealth of diverse sequence information in the metagenome databases which have been used by DeepMSA2. Thus, to make a fair comparison with DeepMSA2, for BLAST, HMMER, MMseqs2, and PSIBLAST, we utilized a combined database of Uniref90[7] and ColabFold-env[8], which covers all of the third-party genomics/metagenomics datasets (including UniProt[7], Metaclust[9], BFD[9], and Mgnify[10]) used in DeepMSA2. For HHblits, which requires a pre-built Hidden Markov Model (HMM) formatted database, the UniRef30[6] and BFD HHblits-style databases were downloaded directly from the HHblits website and utilized to generate MSAs.

For MMseqs2, the default pipeline was the ColabFold[8] MMseqs2 pipeline ("colabfold_search seq.fasta $db"). For the other four control methods (BLAST, HHblits, HMMER, and PSIBLAST), the following commands were employed: "blastall -p blastp -b 100000 -e 0.001 –I seq.fasta -d $db" for BLAST, "hhblits3 -I seq.fasta -d $db -id 99 -cov 50 -n 3 -diff inf -e 1" for HHblits, "jackhammer -N 3 -E 1e-4 –incE 1e-4 seq.fasta $db" for HMMER, and "blastpgp -b 100000 -j 3 -e 0.001 -h 0.001 -d $db -I seq.fasta". In these commands, "$db" represents the path to the databases that combine Uniref90 and ColabFold-env.

The resultant MSAs were utilized to calculate various metrics, including the number of effective sequences (*Neff*), average sequence identity (*SeqId*), and alignment coverage (*cov*). Additionally, to evaluate their ability to capture evolutionary and co-evolutionary information, we further examined the performance of the MSAs in template recognition and deep-learning spatial restraint prediction tests. For the template recognition test, HHsearch[11] is employed, utilizing the profile Hidden Markov Model (HMM) derived from the six sets of MSAs as input. The PDB70 template library that was built before May 1, 2022, was used for HHsearch. Thus, for CASP15 domains, none of the solved experimental structure were included in this template library. For CASP13 and CASP14 domains, to remove the effect of experimental structure and close homologous structure, all homologous templates with a sequence identity >30% to the target were excluded. For the deep learning spatial restraint prediction test, we employed the DeepPotential program[12], utilizing the six sets of MSAs directly as input, and calculate both precision of the top L long-range contacts and *MAE* of the top 5L long-range distances as measures for comparison between MSA generation methods.

In **Figs 2 and S2**, we show the above characteristics of the MSAs provided by DeepMSA2 and the five control pipelines: BLAST[1], HHblits[2], HMMER[3], MMseqs2[4], and PSIBLAST[5]. In **Fig S2A-C**, we first list the comparison results on the number of effective sequences (*Neff*, defined by **Eq. 1** in **Methods** of main text), the average sequence identity (*SeqId*), and alignment coverage (*cov*) between the query and homologous sequences of the MSAs, for the 293 domains from the CASP13-15 experiments. We note that from this set of domains, 161 are template-based modeling (TBM) and 132 sequences are free modeling (FM) domains, where the former have homologous templates in the PDB but the latter do not. The mean *Neff* of the MSAs obtained using DeepMSA2 (415.3) is considerably higher than those from BLAST (120.9) and MMseqs2 (172.4), indicating a reasonable ability of DeepMSA2 to detect sequence homologies. However, the *Neff* of DeepMSA2 is lower (or significantly lower) than those of HHblits (444.9), PSIBLAST (858.4) and HMMER (3377.3). A closer look at the data shows that the sequence alignments from these three control methods (especially HMMER) have a much lower *cov*, probably because they are mainly built on local sequence alignments that do not fully cover the query. In addition, we noticed that MSAs obtained using BLAST, MMseqs2, and PSIBLAST have a much higher *SeqId* than those of

DeepMSA2, HHblits, and HMMER, showing that the former group of methods tend to pick up more similar sequences to the query and therefore produce less diverse MSAs (**Table S4**).

Nevertheless, the parameters considered above (*Neff*, *SeqId*, *cov*) only measure general aspects of the alignment information of the MSAs, and do not necessarily reflect the inherent evolutionary and co-evolutionary information contained in the MSAs (**Fig S3**), which are critical to deep learning-based protein structure predictions. As a more direct test of the ability of each MSA generation method to capture evolutionary and co-evolutionary information, we further examine the performance of the MSAs in assisting template recognition and deep learning spatial restraint prediction. In **Fig 2A**, we list the average TM-scores of the structure templates recognized by HHsearch based on the profile HMMs constructed from the six different MSAs. It is shown that the templates detected using the DeepMSA2 MSA have the highest TM-score for both FM and TBM domains. The average TM-score for all CASP domains obtained using the MSA from DeepMSA2 (0.492) is also higher than those using the MSAs from BLAST (0.454), MMseqs2 (0.469), HHblits (0.463), HMMER (0.448), or PSIBLAST (0.448), all with *p*-values <2.72E-08 by one-sided Student's t-test (**Table S1**).

In **Fig 2B**, we present the precision of the top *L* long-range contact predictions made by the deep neural-network program DeepPotential[12], using co-evolutionary features derived from the six different MSAs (where L is the query sequence length, and "long-range" represents a sequence separation $|i-j| \geq 24$ residues for the contacts between residues *i* and *j*, which are then ranked by the DeepPotential contact probability). Again, utilizing the DeepMSA2 MSA results in a higher precision of top *L* long-range contacts (=0.601) predicted by DeepPotential, compared to those obtained while using the MSAs from BLAST (=0.514), MMseqs2 (0.568), HHblits (0.559), HMMER (0.538), or PSIBLAST (0.566) as inputs for DeepPotential. A similar tendency can be seen for top *L*/5 and *L*/2 predictions as detailed in **Table S2**, where *p*-values are below 1.23E-05 for all the comparisons when all domains in our evaluation set are considered.

In **Fig 2C**, we further display the mean absolute distance error (*MAE*; see **Eq. 6** in **Methods** of main text) of the top 5*L* long-range distances predicted by DeepPotential, where the use of the MSA from DeepMSA2 results in an *MAE*=2.22Å, which is significantly lower than those from the other five MSA programs, i.e., 3.09Å (*p*-value=1.62E-22) for BLAST, 2.70Å (*p*-value=5.79E-12) for PSIBLAST, 2.68Å (*p*-value=1.03E-07) for MMseqs2, 2.74Å (*p*-value=1.26E-09) for HHblits, and 2.98Å (*p*-value=4.80E-13) for HMMER (**Table S3**). In **Fig S1**, we also display a head-to-head comparison of the *MAE* between DeepMSA2 and the five control methods, where DeepMSA2 has lower *MAE* values than the other MSA methods for a dominant fraction of the domains; this accounts for the major reason for the significant improvements observed with DeepMSA2. Overall, these data show that the balanced and highly informative MSA construction provided by DeepMSA2 might have encoded more relevant co-evolutionary features and help guide accurate template recognition and spatial restraint predictions; this ability is also important for the subsequent deep learning-based tertiary structure prediction.

Text S2. Cases analyses of the CASP15 nanobody-antigen complexes

Nanobodies are single-domain antibodies that initiate critical immune reactions by interacting with antigens[13]. In **Fig S7**, we show three illustrative examples from targets H1140, H1141, and H1144, which represent three typical interaction modes of nanobodies with the same mouse CNPase. As illustrated in **Fig S7**, the complex models generated by AlphaFold2-Multimer (from the Elofsson lab's 'NBIS-AF2-multimer' group) exhibit relatively low TM-scores below 0.7. In contrast, DMFold-Multimer has demonstrated exceptional predictive capabilities, achieving TM-scores of 0.92, 0.95, and 0.99, respectively. Consequently, the ICS F1 scores associated with the DMFold-Multimer models (0.51, 0.79, and 0.74) far surpass those of AlphaFold2-multimer (0.02, 0.06, and 0.09). This observation indicates the correct construction of the DeepMSA2 multimer MSAs contributes to the substantial enhancement in the modeling of quaternary chain interactions in these immune protein-antigen complex targets.

In **Fig S8**, we take Target H1144 as an example to further examine the possible reason for the successful modeling by DMFold-Multimer compared to AlphaFold2-Multimer on the nanobody-antigen complexes. First, **Fig S8A** shows a 3D scatter plot of the TM-score, predicted TM-score, and *Neff* of paired MSAs for

the structural decoys by DMFold-Multimer. Here, DMFold-Multimer utilized 25 paired MSAs created by DeepMSA2 which have *Neff*s ranging from 1.8 to 16.3 and created 625 decoy conformations in which 13.6% have high quality with TM-score above 0.8. Importantly, there is a decent correlation between the actual TM-score and predicted TM-score in the high TM-score region (see the top-right area of the 2D TM-score vs predicted TM-score plane of **Fig S8A**), which allows DMFold-Multimer to select a correct model with TM-score 0.99 based on the predicted TM-score. It is notable that this best model comes from the MSA with the highest *Neff* (=16.3) that contains more abundant and relevant co-evolutionary information for quaternary structure modeling.

Here, we note that although the nanobody and the antigen came from different species (i.e., alpaca and mouse) and there may not be evolutionary signals in the inter-species complexes, clear co-evolutionary signal could still be obtained in the DeepMSA2 paired MSAs, since the paired sequences of component MSAs are selected from the same species, which could be used to assist nanobody-antigen complex structure predictions (see "**DeepMSA2-Multimer pipeline for multimeric MSA construction**" section of the **Methods** in the main text). Indeed, we found that even though the nanobody itself is the product of adaptive immune molecule maturation, the DeepMSA2 MSAs provided information on how Ig-like folds can interact with folds resembling the target protein in other species, contributing to the quality of the resulting model. For the case of H1144, DeepMSA2 identified 413 paired homologous sequences that came from 172 common species (**Fig S8C**), where the co-evolution information contained in the paired sequences helps the deep learning networks learn the inter-chain distance restraints, resulting in an accurate predicted distance map (**Fig S8B**).

As a comparison, **Fig S8D** displays the 3D scattering plot for the decoys by re-running AlphaFold2-Multimer, which utilized a single MSA with *Neff*=8.1 and generated no models with TM-score above 0.8. In **Figs S8B** and **8E**, we compare the distance map restraints for the models with the highest predicted TM-scores by DMFold-Multimer and AlphaFold2-Multimer, respectively. Although both programs have correct distance predictions for the intra-chain residues, only DMFold-Multimer has a correct distance map between the inter-chain residues (with a low *MAE*=0.61 Å, compared to 4.35 Å by AlphaFold2-Multimer), which is essential for correct quaternary structure modeling. This example highlights the advantage of DMFold-Multimer in utilizing a multiple MSA pairing strategy and linking sequences from the common species to extract diverse co-evolutionary information, which covers a more extensive quaternary conformational space. In the presence of that improved co-evolutionary information, we observe that the positive TM-score and predicted-TM-score correlation allows for the selection of correct complex models.

Nevertheless, DMFold-Multimer could not fold all the nanobody-antigen complexes in CASP15. In **Fig S9**, we present the modeling result on Target H1142, the only case out of the five nanobody-antigen targets in CASP15 for which DMFold-Multimer had a predicted model with a TM-score below 0.90 (**Fig S9A**). The TM-score of DMFold-Multimer model is 0.98 for H1143 which was not shown in **Fig S7**; see https://www.predictioncenter.org/casp15/multimer_results.cgi?target=H1143. Although DMFold-Multimer also uses multiple paired MSAs with *Neff* ranging from 2.0 to 17.0 for this target (**Fig S9B**), none of the MSAs contains correct inter-chain co-evolutionary information or created correct inter-chain distance maps as shown in **Fig S9C**. As a result, all the created conformational decoys have low quaternary TM-scores (**Fig S9B**), and the final model selected using predicted TM-score has thus a completely incorrect inter-chain orientation with a poor TM-score 0.614, despite the correct tertiary folding in the individual chains (**Fig S9A**). Interestingly, the maximum of predicted TM-scores for H1142 ($pTMS_{max}$=0.75) is considerably lower than that of all other successfully folded nanobody-antigen complexes, i.e., $pTMS_{max}$=0.82, 0.88, 0.90 and 0.90 for H1140, H1141, H1143, and H1144, respectively, suggesting that the $pTMS_{max}$ may be used as a potential indicator for estimating the quality of the DMFold-Multimer models in blind prediction experiments.

References

1. Altschul, S.F., Gish, W., Miller, W., Myers, E.W. & Lipman, D.J. Basic local alignment search tool. *J Mol Biol* **215**, 403-410 (1990).
2. Remmert, M., Biegert, A., Hauser, A. & Soding, J. HHblits: lightning-fast iterative protein sequence searching by HMM-HMM alignment. *Nat Methods* **9**, 173-175 (2012).
3. Eddy, S.R. Accelerated Profile HMM Searches. *PLoS Comput Biol* **7**, e1002195 (2011).
4. Steinegger, M. & Soding, J. MMseqs2 enables sensitive protein sequence searching for the analysis of massive data sets. *Nat Biotechnol* **35**, 1026-1028 (2017).
5. Altschul, S.F. et al. Gapped BLAST and PSI-BLAST: a new generation of protein database search programs. *Nucleic Acids Res* **25**, 3389-3402 (1997).
6. Mirdita, M. et al. Uniclust databases of clustered and deeply annotated protein sequences and alignments. *Nucleic Acids Res* **45**, D170-D176 (2017).
7. Suzek, B.E. et al. UniRef clusters: a comprehensive and scalable alternative for improving sequence similarity searches. *Bioinformatics* **31**, 926-932 (2015).
8. Mirdita, M. et al. ColabFold: making protein folding accessible to all. *Nat Methods* **19**, 679-682 (2022).
9. Steinegger, M. & Soding, J. Clustering huge protein sequence sets in linear time. *Nat Commun* **9**, 2542 (2018).
10. Richardson, L. et al. MGnify: the microbiome sequence data analysis resource in 2023. *Nucleic Acids Res* **51**, D753-D759 (2023).
11. Soding, J. Protein homology detection by HMM-HMM comparison. *Bioinformatics* **21**, 951-960 (2005).
12. Li, Y. et al. Protein inter-residue contact and distance prediction by coupling complementary coevolution features with deep residual networks in CASP14. *Proteins* (2021).
13. Muyldermans, S. Nanobodies: natural single-domain antibodies. *Annu Rev Biochem* **82**, 775-797 (2013).
