# Supplementary material for: Improving deep learning protein monomer and complex structure prediction using DeepMSA2 with huge metagenomics data
Source: Nat Methods. 2024 Jan 2;21(2):279–89. doi: 10.1038/s41592-023-02130-4 (PMC10864179; doi:10.1038/s41592-023-02130-4)
Supplement: Supplementary file 2 — Reporting Summary [file 41592_2023_2130_MOESM2_ESM.pdf]

nature portfolio

Corresponding author(s): Yang Zhang

Last updated by author(s): Oct 6, 2023



# Reporting Summary

Nature Portfolio wishes to improve the reproducibility of the work that we publish. This form provides structure for consistency and transparency in reporting. For further information on Nature Portfolio policies, see our Editorial Policies and the Editorial Policy Checklist.

## Statistics

For all statistical analyses, confirm that the following items are present in the figure legend, table legend, main text, or Methods section.

| n/a | Confirmed | |
|---|---|---|
| ☐ | ☒ | The exact sample size (*n*) for each experimental group/condition, given as a discrete number and unit of measurement |
| ☐ | ☒ | A statement on whether measurements were taken from distinct samples or whether the same sample was measured repeatedly |
| ☐ | ☒ | The statistical test(s) used AND whether they are one- or two-sided<br>*Only common tests should be described solely by name; describe more complex techniques in the Methods section.* |
| ☒ | ☐ | A description of all covariates tested |
| ☐ | ☒ | A description of any assumptions or corrections, such as tests of normality and adjustment for multiple comparisons |
| ☐ | ☒ | A full description of the statistical parameters including central tendency (e.g. means) or other basic estimates (e.g. regression coefficient) AND variation (e.g. standard deviation) or associated estimates of uncertainty (e.g. confidence intervals) |
| ☐ | ☒ | For null hypothesis testing, the test statistic (e.g. *F*, *t*, *r*) with confidence intervals, effect sizes, degrees of freedom and *P* value noted<br>*Give P values as exact values whenever suitable.* |
| ☒ | ☐ | For Bayesian analysis, information on the choice of priors and Markov chain Monte Carlo settings |
| ☒ | ☐ | For hierarchical and complex designs, identification of the appropriate level for tests and full reporting of outcomes |
| ☒ | ☐ | Estimates of effect sizes (e.g. Cohen's *d*, Pearson's *r*), indicating how they were calculated |

*Our web collection on statistics for biologists contains articles on many of the points above.*

## Software and code

Policy information about availability of computer code

| | |
|---|---|
| Data collection | No software was used to collect benchmark dataset. All data are free available on CASP13, CASP14 and CASP15 sub-sections at https://www.predictioncenter.org/index.cgi. MEGAHIT (1.0), Prodigal (2.6), CD-HIT (4.6), FragGeneScan (1.2), and MMseqs2 (version aa175d63658d9aa2e908325a6fd40e9dbb260c9a) were used to collect TaraDB, MetaSourceDB and JGIclust metagenomics databases. |
| Data analysis | The MSAs and structure models were generated by DeepMSA2 (2.0) server (https://zhanggroup.org/DeepMSA2/) and DMFold server (https://zhanggroup.org/DMFold/), and all statistical analyses were done by R (4.1.2) software. AlphaFold2 (2.2.0) was used as control method for checking the modeling quality of protein monomer and multimer. DeepPotential (1.0) was used to generate contact and distance restraints for CASP13-15 protein monomers. HHblits (2.0.15 and 3.1.0), HMMER (3.1b2), BLAST (2.2.26), kClust (1.0), Clustal Omega (1.2.4), and AlphaFold2 (2.2.0) has been used to generate and rank MSAs in DeepMSA2 method. US-align (Version 20220626) was used to analysis the model quality. |

For manuscripts utilizing custom algorithms or software that are central to the research but not yet described in published literature, software must be made available to editors and reviewers. We strongly encourage code deposition in a community repository (e.g. GitHub). See the Nature Portfolio guidelines for submitting code & software for further information.

## Data

Policy information about availability of data

All manuscripts must include a data availability statement. This statement should provide the following information, where applicable:

- Accession codes, unique identifiers, or web links for publicly available datasets
- A description of any restrictions on data availability
- For clinical datasets or third party data, please ensure that the statement adheres to our policy

Third-party databases, Uniclust30 (UniRef30), Uniref90, Metaclust, BFD, MGnify are used this work, those databases are available at https://gwdu111.gwdg.de/~compbiol/uniclust/, https://ftp.uniprot.org/pub/databases/uniprot/current_release/uniref/uniref90/, https://metaclust.mmseqs.org/, https://bfd.mmseqs.com/, and http://ftp.ebi.ac.uk/pub/databases/metagenomics/peptide_database/, respectively. All CASP benchmark data used in this work are available at https://zhanggroup.org/DMFold/ or https://zenodo.org/record/8371924 (https://doi.org/10.5281/zenodo.8371924). The structure modeling results on 5,042 human proteome proteins are freely available at https://zhanggroup.org/DMFold/human for academic use. Source data are provided with this paper.

## Human research participants

Policy information about studies involving human research participants and Sex and Gender in Research.

| | |
|---|---|
| Reporting on sex and gender | NA |
| Population characteristics | NA |
| Recruitment | NA |
| Ethics oversight | NA |

Note that full information on the approval of the study protocol must also be provided in the manuscript.

# Field-specific reporting

Please select the one below that is the best fit for your research. If you are not sure, read the appropriate sections before making your selection.

☒ Life sciences ☐ Behavioural & social sciences ☐ Ecological, evolutionary & environmental sciences

For a reference copy of the document with all sections, see nature.com/documents/nr-reporting-summary-flat.pdf

# Life sciences study design

All studies must disclose on these points even when the disclosure is negative.

| | |
|---|---|
| Sample size | The manuscript includes an evaluation of structure predictions for 293 protein monomer targets from CASP13, CASP14 and CASP15, and 54 protein complex targets from CASP13 and CASP14. 38 protein complex targets from the CASP15 blind test are also used in the manuscript. 5,042 human proteins where AlphaFold2 has bad-quality models are modeled by DMFold, and the query sequences are taken from UniProt. No statistical method was used to decide the sample size, but the number of the samples is sufficient for applied statistical analysis in each case. In addition, the CASP datasets were taken from the community-wide experiments, where all datasets are standard testing sets in this field. |
| Data exclusions | The proteins homologous (based on the release date) to the benchmark dataset were excluded from the template library to avoid homologous contamination. Six CASP domains are excluded from threading benchmark test because HHsearch failed to generate results with some MSAs from third-party control methods (i.e., HMMER MSAs contain too many sequences). 22 CASP domains are excluded from DeepPotential restraint prediction benchmark test because DeepPotential failed to generate results with some MSAs from third-party control methods (i.e., HMMER MSAs contain too many sequences), or some targets are extremely hard for DeepPotential to make a prediction (i.e., MAE>30 for all MSAs). Based on simple statistic analysis (i.e., average value), excluding those data dose not change the conclusion that DeepMSA2 performs better than the five control methods. |
| Replication | All results could be reproduced by our server and standalone package with the full version databases, or based on the information provided in manuscript. All experiments are done independently without any technical replication. |
| Randomization | There is no random method used in the manuscript. All data in CASP13-15 are used in the manuscript. Covariants are not relevant in this study since the CASP benchmark dataset is the blind and golden standard benchmark dataset in benchmarking protein structure prediction, and most of the 'FM' targets in CASP datasets do not have homologous structure in PDB, and thus are not redundant with others. |
| Blinding | There was no blinding group or benchmarking analysis on CASP13 and CASP14 datasets in this manuscript. The CAPS15 results are taken from CASP official website, and the authors participated the CASP15 world-wide experiment test with blinding, where all experimental structures |

were not available to the authors during the CASP15 season; thus, results from CASP15 represent a community-wide standard blind experiment.

# Reporting for specific materials, systems and methods

We require information from authors about some types of materials, experimental systems and methods used in many studies. Here, indicate whether each material, system or method listed is relevant to your study. If you are not sure if a list item applies to your research, read the appropriate section before selecting a response.

## Materials & experimental systems

| n/a | Involved in the study |
|---|---|
| ☒ | ☐ Antibodies |
| ☒ | ☐ Eukaryotic cell lines |
| ☒ | ☐ Palaeontology and archaeology |
| ☒ | ☐ Animals and other organisms |
| ☒ | ☐ Clinical data |
| ☒ | ☐ Dual use research of concern |

## Methods

| n/a | Involved in the study |
|---|---|
| ☒ | ☐ ChIP-seq |
| ☒ | ☐ Flow cytometry |
| ☒ | ☐ MRI-based neuroimaging |
